# Elevated hemoglobin A1c is associated with the presence of pancreatic cysts in a high-risk pancreatic surveillance program

**DOI:** 10.1186/s12876-020-01308-w

**Published:** 2020-05-27

**Authors:** Ariel Bar-Mashiah, Anne Aronson, Monica Naparst, Christopher J. DiMaio, Aimee L. Lucas

**Affiliations:** Henry D. Janowitz Division of Gastroenterology, Icahn School of Medicine at Mount Sinai, New York, USA

**Keywords:** Type 3c diabetes, Pancreatic diabetes, Early detection, Pancreatic cysts

## Abstract

**Background:**

Emerging evidence demonstrates that surveillance of individuals at high-risk (HRIs) of developing pancreatic adenocarcinoma allows for identification and treatment of resectable tumors with improved survival. Population-based data suggest that hyperglycemia may be present up to three years before the development of pancreatic cancer. We investigated whether elevated hemoglobin A1c (HbA1c) is associated with the development of pancreatic cysts in a pancreatic surveillance program.

**Methods:**

We performed a retrospective study of HRIs who underwent pancreatic surveillance at a single institution between May 2013 and March 2019, according to published criteria. We collected demographic information, clinical data including HbA1c, and imaging results. We compared data using univariable and multivariable analyses. Our primary outcome was the presence of pancreatic cysts on initial surveillance in patients with elevated HbA1c.

**Results:**

Ninety-eight patients underwent surveillance imaging via EUS or MRCP and seventy-four patients met inclusion criteria. Thirty patients were found to have cysts on initial imaging. Older age (*p* < 0.01) and HbA1c in the prediabetic range or higher (*p* = 0.01) were associated with the presence of cysts or solid lesions on univariable analysis. After controlling for confounders, age (aOR 9.08, 95% CI 2.29–36.10), and HbA1c > 5.7% (aOR 5.82, 95% CI 1.50–22.54) remained associated with presence of cysts and solid lesions in HRIs. In patients with cysts or solid lesions there was a strong association between increased age and elevated HbA1c (*p* < 0.01).

**Conclusion:**

HRIs with elevated HbA1c were more likely to have pancreatic cysts compared to individuals with lower HbA1c on initial imaging in a pancreatic surveillance program. These findings may help tailor the surveillance protocols for those at increased risk of developing pancreatic adenocarcinoma.

## Background

Pancreatic ductal adenocarcinoma is the fourth leading cause of cancer death in the United States, with approximately 56,770 people projected to be diagnosed with pancreatic cancer and 45,750 deaths expected in 2019, and is expected to be the second leading cause of cancer death after 2020 [1, 2]. The five-year survival rate for pancreatic adenocarcinoma remains low at approximately 9%. An international consortium of experts recommended pancreatic surveillance for high-risk individuals (HRIs) with an estimated lifetime risk of > 5% [3–10]. Recent studies have found a benefit to high-risk pancreatic surveillance programs, with 3-year survival rates of 85% and a higher proportion of resectable malignant lesions [11].

Approximately one-third of HRIs have small, sub-centimeter pancreatic cysts on surveillance imaging [12]. The majority of these lesions remain stable during surveillance; however, features such as the presence of multifocal cysts, cyst growth, and the presence of a deleterious germline mutation have been associated with neoplastic progression to high-grade dysplasia or pancreatic cancer [11, 13].

New-onset diabetes mellitus has been established as a prelude to pancreatic adenocarcinoma [14]. Approximately 85% of patients at pancreatic cancer diagnosis have impaired fasting blood glucose or diabetes mellitus, suggesting this may be a near universal marker for progression to pancreatic adenocarcinoma and can aid in its diagnosis during the disease’s earlier stages [15]. Furthermore, elevations in fasting blood glucose may be seen up to 36 months prior to pancreatic adenocarcinoma diagnosis [16].

The aim of our study is to identify whether there is an association between elevated Hemoglobin A1c (HbA1c) and the presence of pancreatic cysts in a high-risk pancreatic surveillance program.

## Methods

### Cohort recruitment

From May 2013 to March 2019 subjects were enrolled in a high-risk registry at a tertiary academic medical center with a comprehensive multidisciplinary pancreas program. The registry and this study are institutional review board approved, and all subjects provided informed written consent. HRIs were included if they met International Cancer of the Pancreas Screening (CAPS) criteria or American College of Gastroenterology (ACG) clinical guidelines for hereditary gastrointestinal cancer (Table 1) [4, 17, 18]. HRIs were referred to the registry via physicians, genetic counselors, and/or self-referral. After providing informed consent, all patients completed a survey that included questions relating to personal medical history, family history, known genetic mutations, social history and environmental exposures such as tobacco and alcohol use history.

**Table 1:** Candidates for Pancreatic Cancer Surveillance

Criteria	Age at Inclusion
FDR of affected family member in FPC kindred	≥ 50 years or 10 years younger than youngest affected family member
*STK11* mutation (Peutz-Jeghers syndrome)	≥ 40 years
*BRCA1, BRCA2, PALB2, ATM*, *MLH1*, *MSH2*, *MSH6* mutation with affected FDR or SDR	≥ 50 years or 10 years younger than youngest affected family member
*CDKN2A*	≥ 40 years
*PRSS1* mutation (hereditary pancreatitis)	≥ 40 years

*FDR* first-degree relative, *FPC* Familial Pancreatic Cancer, defined as kindreds with 2 affected FDRs, *SDR* second-degree relative

### Surveillance protocol

HRIs were offered initial surveillance via endoscopic ultrasound (EUS) and/or magnetic resonance imaging with cholangiopancreatography (MRCP) at least annually, in accordance with existing guidelines [4, 17, 18]. Patients who met criteria for pancreatic cancer surveillance (Table 1), had abdominal imaging with either EUS or MRCP, and HbA1c levels in medical record within one year of office visit were included in this analysis. If patients had multiple HbA1c levels within one year of an office visit, we used the value collected closest to the office visit date.

**Fig. 1:**
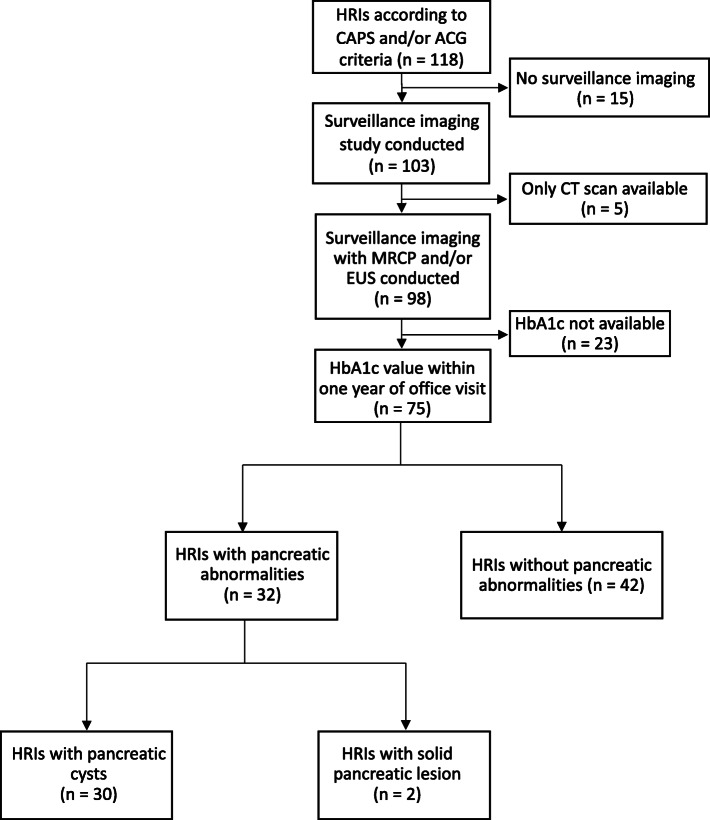
HRIs in our high-risk registry meeting inclusion and exclusion criteria HRIs, high-risk individuals; CAPS, Cancer of the Pancreas Screening Consortium; ACG, American College of Gastroenterology; CT, computed tomography; MRCP, magnetic resonance cholangiopancreatography; EUS, endoscopic ultrasound; HbA1c, Hemoglobin A1c

MRCP was conducted using a GE Signa HDxt 1.5T machine. EUS was conducted and interpreted by one experienced therapeutic endoscopist using an Olympus GF-UCT180 Curvilinear Array Ultrasound Echoendoscope. Five patients had their imaging conducted at an outside institution; however, all relevant medical records were available for review. Imaging data were extracted directly from the electronic medical record. Only findings from initial surveillance imaging were included in our analysis (Fig. 1).

### Statistical analyses

Statistical analyses were conducted using SPSS version 22.0 software (SPSS, Inc., Chicago, IL) with two-sided significance set at a *p*-value of .05. Categorical variables were summarized using descriptive statistics, with comparisons between groups performed using Pearson chi-square test for univariate analysis. Odds ratios and 95% confidence intervals were calculated using logistic regression adjusting for covariates. Cochran-Armitage trend test was conducted using XLSTAT (Microsoft Excel version 16.25) with two-tailed *p*-value set at .05.

### Covariates

Older age was considered ≥ 60 years. Patients were considered overweight or obese if body mass index (BMI) ≥ 25.0 in accordance with Centers for Disease Control and Prevention guidelines. Tobacco use was considered in former and current smokers compared to never smokers. Elevated HbA1c was > 5.7%, which is the clinical criteria for prediabetes.

## Results

Ninety-eight patients underwent pancreatic surveillance by EUS or MRCP. Seventy-four subjects met inclusion criteria: 45 (60.8%) were female, 62 (83.8%) identified as white/Caucasian, 6 (8.1%) as Black, and 4 (5.4%) as Hispanic/Latino (Table 2). Eight patients (10.8%) were found to have a pathogenic/likely pathogenic (P/LP) variant in *BRCA1*, while 13 (17.6%) carried a P/LP variant in *BRCA2*. One patient had a P/LP variant in *ATM* (1.4%) and 2 patients (2.7%) had Lynch Syndrome. All patients with a germline P/LP variant had at least one first-degree or second-degree relative with pancreatic adenocarcinoma. Fifty (67.5%) patients had familial pancreatic cancer (FPC), defined as individuals with 2 or more blood relatives with pancreas cancer, at least 1 of whom was a first-degree relative of the patient, in the absence of a known P/LP germline variant that has been associated with pancreatic adenocarcinoma.

**Table 2:** Characteristics of the study cohort

Variables	n (%)
Total	74 (100.0)
Age	
≤ 40	2 (2.7)
41–50	2 (2.7)
51–60	23 (31.1)
61–70	28 (37.8)
71–80	17 (23.0)
> 80	2 (2.7)
Sex	
Male	29 (39.2)
Female	45 (60.8)
Race	
Asian	1 (1.4)
Pacific Islander	1 (1.4)
Black/African American	6 (8.1)
White/Caucasian	62 (83.8)
Unknown/Other/Prefer Not to Answer	4 (5.3)
Ethnicity	
Hispanic/Latino	4 (5.4)
Not Hispanic/Latino	70 (94.6)
Body Mass Index	
Healthy (< 25.0)	27 (36.5)
Overweight (25.0–29.9)	37 (50.0)
Obese (≥ 30.0)	10 (13.5)
Tobacco Use	
Current Smokers	3 (4.1)
Former Smokers	30 (40.5)
Never Smokers	41 (55.4)
Surveillance Categories	
Familial Pancreas Cancer	50 (67.5)
*BRCA*1 w/ FH	8 (10.8)
*BRCA*2 w/ FH	13 (17.6)
Lynch Syndrome w/ FH	2 (2.7)
*ATM* w/ FH	1 (1.4)
Hemoglobin A1c	
> 5.7%	19 (25.7)
≤ 5.7%	55 (74.3)
Mean Follow Up Time in Years (SD)	2.6 (SD 1.7)

*FH* family history, *SD* standard deviation

Forty-seven (63.5%) patients were older than 60 years. Nineteen patients (25.7%) had HbA1c values greater than 5.7%. Thirty-three (44.6%) subjects identified as former or current smokers. Twenty-seven (36.5%) patients were in the healthy BMI range (< 25.0), 37 (50.0%) were overweight (25.0–29.9) and 10 (13.5%) patients were obese (≥ 30).

The mean follow-up time in clinic was 2.6 years (range 0.2–6.1 years). Thirty patients were found to have pancreatic cysts on initial imaging. The clinical impression by the treating physicians at our institution (ALL, CJD) is that the majority of the cysts were consistent with sub-centimeter branch duct intraductal papillary mucinous neoplasms (IPMNs), although we do not have tissue confirmation of cyst subtypes for most patients. A few cysts did exhibit worrisome features: 2 patients had main pancreatic duct diameter between 5 and 9 mm, 2 cysts grew > 5 mm within a two-year period, 1 cyst had a 3 mm nodule, and 2 cysts were ≥ 3 cm [19]. One subject had both a cyst > 3 cm which exhibited growth of > 5 mm or greater in a two-year period; this patient remains in surveillance. One patient had both a cyst > 3 cm with a 4 mm nodule; upon resection this demonstrated IPMN with high grade dysplasia. A total of five patients had worrisome features; of those patients, three (60%) exhibited HbA1c > 5.7%. Other features noted were 22 patients with multifocal cysts, 22 patients with sub-centimeter cysts, and 2 patients with solid lesions found on initial imaging (Table 3). One patient with a solid lesion had an elevated HbA1c and was found to have pancreatic cancer. The other patient remains in surveillance, and the solid lesion was not redemonstrated on subsequent imaging.

**Table 3:** Pancreatic Abnormalities Found in HRIs on Initial Imaging

Abnormality Detected	N (%)
IPMNs^a^	30 (40.5)
Sub-centimeter cysts	22 (29.7)
Multifocal cysts	22 (29.7)
Worrisome Features^b^	5 (6.8)
Cysts > 3 cm	2 (2.7)
Cyst with solid component	1 (1.4)
MPD 5–9 mm	2 (2.7)
Solid lesions	2 (2.7)

*IPMN* intraductal papillary mucinous neoplasm, *MPD* main pancreatic duct

^a^The clinical impression by the treating physicians was that all cysts were IPMNs. Due to the sub-centimeter size of the cysts and lack of concerning features, the majority of cysts did not have a pathologic confirmation

^b^Worrisome features are based on the 2017 Revised Fukuoka Consensus Guidelines [19]

Older age (*p* < 0.01) and HbA1c in the prediabetic range or higher (*p* = 0.01) were associated with the presence of pancreatic abnormalities on univariable analysis (Table 4). After controlling for confounders, age ≥ 60 (aOR 8.36, 95% CI 2.09–33.44), and HbA1c > 5.7% (aOR 5.48, 95% CI 1.41–21.23) remained associated with presence of cysts in HRIs. In further analyses, age ≥ 60 (aOR 9.08, 95% CI 2.29–36.10), and HbA1c > 5.7% (aOR 5.82, 95% CI 1.50–22.54) remained associated with presence of cysts or solid lesions in HRIs (Table 5).

**Table 4:** Univariate Analysis Evaluating Factors Associated with Pancreatic Abnormalities

Independent Variable	Patients with Cysts or Solid Lesions (*n* = 32; 43.2%)	Patients Without Cysts or Solid Lesions (*n* = 42; 56.8%)	*P* value
Age ≥ 60	28 (87.5)	21 (50.0)	<.01
Female Sex	21 (65.6)	24 (57.1)	.46
HbA1c > 5.7%	13 (40.6)	6 (14.3)	.01
BMI ≥ 25	21 (65.6)	26 (61.9)	.74
Current and Former Smokers	17 (53.1)	16 (38.1)	.20
FPC	23 (71.9)	28 (66.6)	.45

*HbA1c* Hemoglobin A1c, *BMI* Body Mass Index, *FPC* Familial pancreatic cancer

**Table 5:** Multivariate Analysis Evaluating Factors Associated with Presence of Pancreatic Cysts or Solid Lesions

Independent Variable	*P* value	aOR	95% C.I. for aOR Lower Upper	
Age ≥ 60	< .01	9.08	2.29	36.10
HbA1c > 5.7%	.01	5.82	1.50	22.54
Current and Former Smokers	.38	1.65	.54	5.05
BMI ≥ 25	.45	.63	.19	2.10
Female Sex	.07	3.17	.90	11.05
FPC	.80	1.17	.35	3.97

*HbA1c* Hemoglobin A1c, *BMI* Body Mass Index, *FPC* Familial pancreatic cancer

**Fig. 2:**
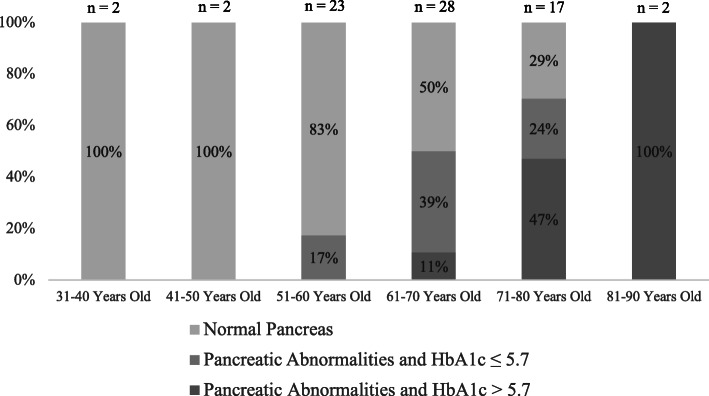
Cochran-Armitage trend test demonstrates that pancreatic cysts or solid lesions are associated with increased age and elevated HbA1c in HRIs (*p* < 0.01)

Patients with cysts or solid lesions were categorized into cohorts according to HbA1c status (> 5.7% v ≤ 5.7%). Cochran-Armitage trend test in patients with cysts or solid lesions showed a significant association between elevated HbA1c and increased age (*p* < 0.01) (Fig. 2).

## Discussion

The results of this study demonstrated an association between elevated HbA1c, age and the presence of pancreatic cysts on initial surveillance imaging in HRIs.

The presence of IPMNs and pancreatic intraepithelial neoplasia (PanINs) indicates a risk of ductal cancer not only in the lesion itself but within the entire pancreas [20]. A majority of patients with cysts in our study had sub-centimeter branch duct IPMNs, which is consistent with prior reports in HRIs [12]. Pancreatic cancer surveillance programs have shown that up to 34% of HRIs ages 50–59 have pancreatic lesions, and this proportion increased to 54% in those ages 60–69 [12]. The majority of these lesions are small (< 1 cm) branch duct IPMNs or PanINs, and are more commonly detected in HRIs than in the general population. Our study supports these data, demonstrating that pancreatic cysts are more commonly identified in older HRIs.

Prior studies have reported an association between older age and increased HbA1c levels in nondiabetic populations (HbA1c < 5.7%) [21]. While our analyses do not argue against an association between increasing HbA1c and age, we focused on individuals at increased risk of pancreatic cancer. We have additionally sought to focus on whether impaired glucose metabolism, evidenced here by HbA1c levels above the prediabetic cutoff, are associated with the presence of pancreatic cysts in a unique cohort of individuals at high-risk of developing pancreatic cancer.

In HRIs, neoplastic progression has been described to occur more commonly in germline mutation carriers and individuals with multifocal cysts [11]. In our study, 22 (29.7%) patients had multifocal cysts and 24 (32.4%) carried P/LP germline variants associated with pancreatic cancer. Mean surveillance in this study was 2.6 years. Therefore, our analyses focused on initial surveillance imaging studies; with a longer surveillance period, we will be able to determine if these factors and HbA1c levels predict neoplastic progression.

Tobacco is a known independent risk factor for pancreatic cancer [22]. A recent study of HRIs has described an association between increased age and smoking history with the development of pre-malignant lesions or early pancreatic adenocarcinoma [23]. Similar to their study, we found that increased age is associated with the development of cysts; however, we did not find that smoking was associated with precursor lesions such as IPMNs and PanINs. However, it is possible that among patients that smoke, pack-year tobacco exposure differed between the European and US-based studies. Further studies on the impact of tobacco exposure in HRIs are needed.

Two major causes of diabetes are pancreatic beta cell dysfunction and peripheral insulin resistance. However, type 3c diabetes is another form of diabetes caused by a variety of pancreatic diseases with varying mechanisms of hyperglycemia [24]. Up to 80% of individuals with pancreatic cancer have evidence of impaired glycemic metabolism, and a diagnosis of new-onset diabetes has been associated with up to an 8-fold increased risk of pancreatic cancer [15, 25]. Elevations in fasting blood glucose may be apparent up to 36 months prior to a diagnosis of pancreatic cancer [16]. Recent reports suggested that FPC kindreds have a similar abnormalities in their glycemic profile prior to the diagnosis of pancreatic cancer compared to those with sporadic pancreatic cancer [26]. In this study, one patient with multifocal cysts went on to progress to adenocarcinoma associated with hyperglycemia. Our study provides additional support for further evaluation of glycemic profiles in HRIs to improve risk stratification [16].

Models to distinguish between pancreatic cancer-induced hyperglycemia and the prediabetes of type 2 diabetes mellitus have found that variables typically associated with increased risk of type 2 diabetes, including elevated BMI, hypercholesterolemia and hypertriglyceridemia, were associated with lower pancreatic adenocarcinoma risk [27]. Others have noted that new-onset diabetes prior to pancreatic cancer development is associated with paradoxical weight loss [28]. Multiple analyses have also demonstrated that obesity is associated with the development of pancreatic cancer [29–31]. In our study, we were not able to draw conclusions between BMI and cyst development in HRIs since the majority (86%) of our patients were found to be healthy or overweight. Future studies should address longitudinal changes in BMI and glycemic profiles in HRIs.

Other studies have evaluated the association between treatment for diabetes and overall survival in patients with pancreatic adenocarcinoma [32]. For example, one study found that metformin, a biguanide oral hypoglycemic commonly used as first-line therapy in patients with diabetes, is associated with increased survival in patients with pancreatic cancer [33]. Since only four of our subjects were found to be in the diabetic range (HbA1c ≥ 6.5%), we were unable to evaluate the impact of diabetes treatment in HRIs.

There are a number of strengths and limitations to this study. Although we assessed the presence of pancreatic cysts in HRIs, the presence of cysts does not suggest that these lesions will eventually progress into cancer. Additionally, as this study was retrospective in nature, we were not able to collect HbA1c and BMI values at the exact date of surveillance imaging. Patients were only included if they had HbA1c testing within one year of a clinical visit. However, one strength of this approach is that HbA1c data did not alter clinical decisions and prompt earlier investigations. We were not able to conduct a subgroup analysis in patients with worrisome features due to the small number of patients that fell into this category. Additionally, we do not have any information regarding differentiation of IPMN subtypes except for one patient who had a surgical resection. Since this study was conducted only in patients with a high risk of developing pancreatic cancer, the results are not generalizable to the general population.

At this time, HRIs with cysts in the setting of elevated HbA1c should continue surveillance under published guidelines [4, 18]. In newly established guidelines, there was expert consensus around the need for glucose testing (fasting glucose or HbA1c) to detect new-onset diabetes in HRIs. Additionally, there was consensus that the new emergence of diabetes in HRIs should prompt additional investigation [4]. Further prospective surveillance data are required to evaluate risk factors for neoplastic progression in HRIs with cysts and elevated HbA1c.

## Conclusion

In conclusion, our study demonstrates that elevated HbA1c is associated with the presence of pancreatic cysts on initial surveillance imaging in HRIs in a high-risk pancreatic surveillance program. Identification of pancreatic abnormalities and other risk factors that predict neoplastic progression to adenocarcinoma or high-grade precursor neoplasms may improve surveillance programs and guide management of detected lesions in the future. Future studies evaluating longitudinal changes in the glycemic and metabolic profiles in HRIs should be pursued.

## Data Availability

The datasets used and/or analyzed during the current study are available from the corresponding author on reasonable request.

## Abbreviations

HRIs: High-risk individuals;
Hemoglobin A1c: HbA1c
EUS: Endoscopic ultrasound
MRCP: Magnetic resonance cholangiopancreatography
BMI: Body mass index
FPC: Familial pancreatic cancer
IPMN: Intraductal papillary mucinous neoplasm
PanIN: Pancreatic intraepithelial neoplasia
FH: Family history
